# Clinical observation of radiofrequency ablation for premature ventricular contractions originating from uncommon His-Purkinje sites

**DOI:** 10.1007/s10840-022-01428-1

**Published:** 2022-11-22

**Authors:** Jing Huang, Yidong Zhao, Long Yang, Qifang Liu

**Affiliations:** Department of Cardiology, Guizhou Provincial People’s Hospital, No 83, ZhongShan East Road, Guiyang, 550002 China

**Keywords:** Ablation, Fascicle, Mapping, Premature ventricular contractions

## Abstract

**Background:**

Radiofrequency catheter ablation (RFCA) of premature ventricular contractions (PVCs) originating from common locations such as the proximal and middle fascicles of the His-Purkinje system (HPS) has been established as an effective therapy. This report aims to highlight the electrophysiological properties and RFCA of PVCs originating from uncommon locations of the HPS.

**Methods:**

Among 57 patients with fascicular PVCs, 3 with fascicular PVCs originating from uncommon sites were retrospectively analyzed.

**Results:**

We identified three patients with PVCs originating separately from diseased fascicles, the dead-end tract (DET), and the distal fascicle. In contrast to PVCs originating from the proximal and medial fascicles, the fascicular potentials could not be recorded at the target sites of patients with PVCs originating from diseased fascicles or the distal fascicle during sinus rhythm. However, these PVCs were successfully ablated from the HPS, guided by recording their earliest fascicular potentials in PVCs. PVCs originating from the DET are morphologically consistent with those originating from the proximal left anterior fascicle or the distal left bundle branch. The corresponding tiny sharp potential of the DET could be mapped, and RFCA of the right coronary cusp achieved successful suppression of PVCs.

**Conclusions:**

The knowledge of the different electrophysiological characteristics of fascicular PVCs originating from uncommon locations can contribute to precise mapping and ablation. For such arrhythmia, the target site for successful ablation should be identified by earliest fascicular potential.

## Introduction

Radiofrequency catheter ablation (RFCA) of premature ventricular contractions (PVCs) originating from the His-Purkinje system (HPS) is developing rapidly, and the strategy for ablation is becoming increasingly sophisticated. With the help of 3D electro-anatomical activation mapping, the earliest fascicular potential (FP) can be mapped, rather than the earliest ventricular myocardial activation, which provides a solid basis for a successful RFCA therapy [1, 2]. Previous studies have focused on the mapping and ablation of PVCs originating from common locations such as the proximal and middle fascicles of the HPS [3, 4]. PVCs originating from diseased fascicles, distal fascicles, and special branches of the fascicles have been rarely reported. This study aimed to investigate the electrophysiological characteristics and RFCA of PVCs originating from these uncommon locations of the fascicles.

## Methods

A total of 57 consecutive patients with PVCs originating from the HPS who underwent catheter ablation at Guizhou Provincial People’s Hospital from January 2018 to February 2021 were included. Written informed consent was obtained from all patients. Structural abnormalities were excluded by echocardiography and coronary angiography or cardiac magnetic resonance, when needed. During the electrophysiologic study, sustained ventricular tachycardia was not inducible in all patients. Electroanatomic mapping was performed using CARTO (Biosense Webster). Activation mapping was performed using a 3.5-mm irrigated tip quadripolar ablation catheter (ThermoCool, Biosense Webster). Bipolar electrograms (filtered at 30 to 400 Hz) were recorded from the distal electrode pair of the ablation catheter. Unipolar electrograms (filtered at 1 to 240 Hz, Wilson’s central terminal) were recorded from the distal electrode of the ablation catheter. Three patients with fascicular PVCs originating from uncommon locations were retrospectively analyzed for electrophysiological mappings and RFCA. The electrophysiology studies and clinical outcomes are reported below.

## Case presentation

### Case 1

**Fig. 1:**
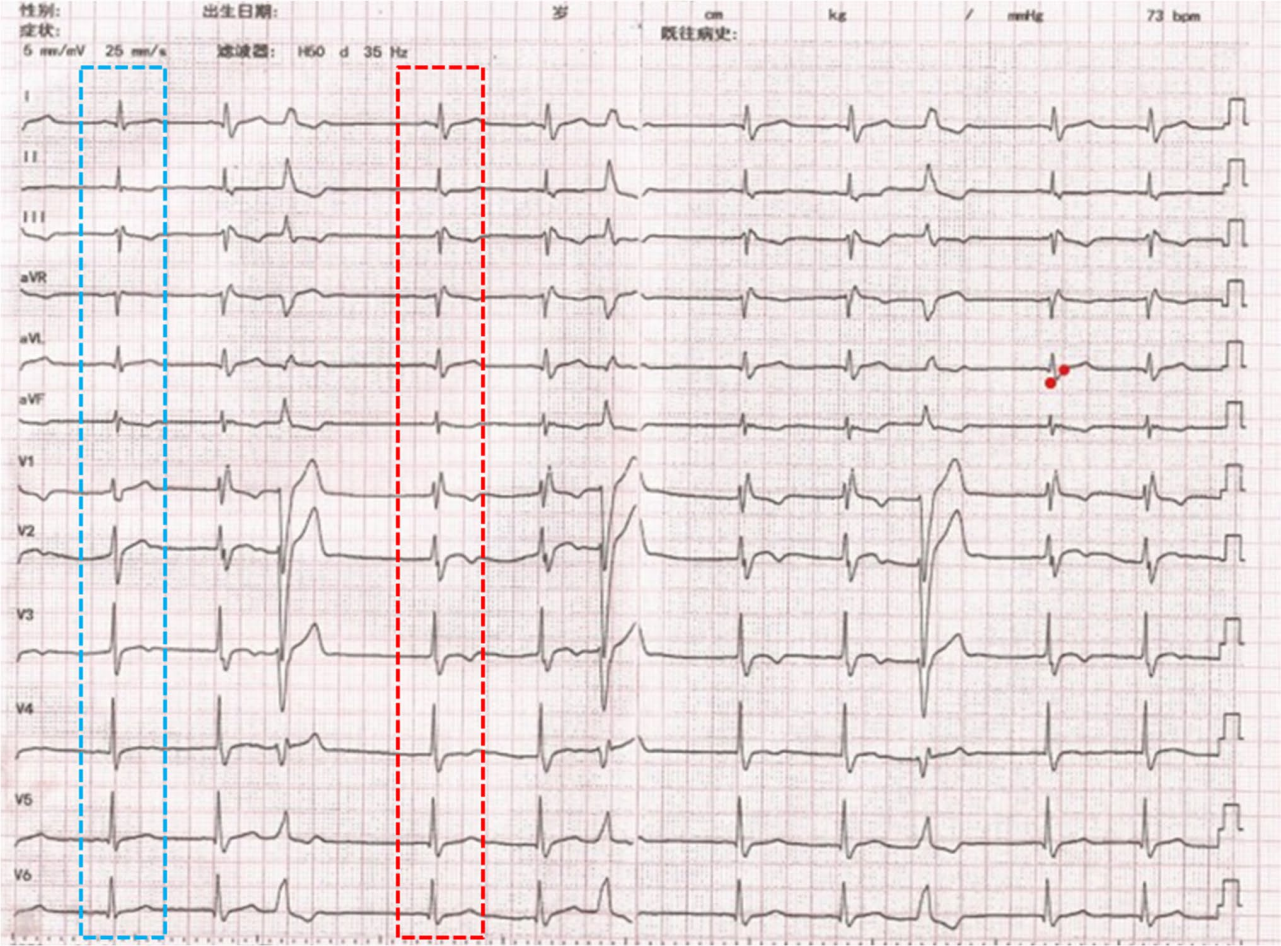
Electrocardiogram shows a sinus rhythm with intermittent RBBB and frequent PVCs. The red dashed box shows complete RBBB and the blue dashed box shows no bundle branch block

**Fig. 2:**
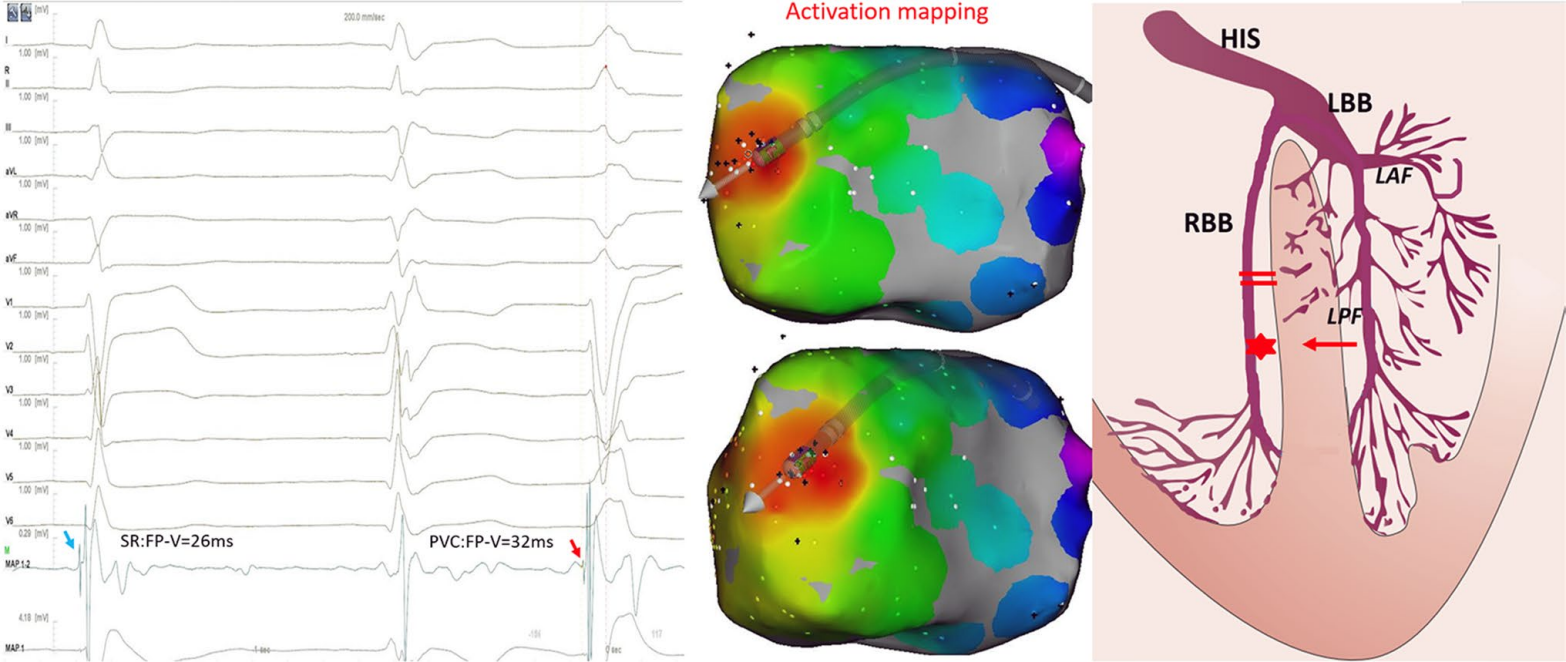
Electro-anatomical activation mapping of the PVCs and model diagrams of origin location of PVCs. Left panel: FP was recorded in sinus rhythm with no RBBB (blue arrow); and no FP was recorded in sinus rhythm with RBBB; FP was recorded in PVCs at earliest activation location (red arrow). Right panel: model diagram of the mechanism without FP in sinus rhythm, the red star represents the origin location of PVCs

A 28-year-old man was referred to our hospital for catheter ablation of previously diagnosed (definite diagnosis) symptomatic frequent PVCs after 3 months of ineffective treatment with standard anti-arrhythmic medication metoprolol and verapamil. The 24-h Holter monitoring showed PVCs of 21,897 and the QRS duration of the PVCs was 137 ms. The anti-arrhythmic medications were discontinued for more than five half-lives before the operation. Baseline 12-lead electrocardiogram (ECG) showed sinus rhythm with intermittent right bundle branch block (RBBB) and frequent PVCs. The morphology of PVCs was characterized by a pattern of left bundle branch block and a transition at V5 (Fig. 1). Activation mapping of PVCs was performed in the right ventricle. A sharp FP that preceded the beginning of the V wave with an isoelectric line between them was recorded at the earliest activation site. The FP was not observed in sinus rhythm with complete RBBB and was visible when the RBBB disappeared (Fig. 2). RFCA was attempted at the earliest activation site in a power-controlled mode at 30 W, causing the PVCs to disappear within 10 s and not recur. Following completion of the lesion, over a time period of 30 min with isoproterenol infusion, no further PVCs were observed. ECGs and 24-h Holter monitoring 3 months after the RFCA revealed sinus rhythm with complete RBBB and no PVCs. Previous studies indicate that the activation mapping of PVCs originating from the fascicles is able to record FPs during sinus rhythm and PVCs at the earliest activation location, which confirms the diagnosis of fascicular PVCs [3]. In this case, the QRS morphology showed a RBBB in sinus rhythm, and the FP failed to be mapped. It was initially considered to be of ventricular myocardial origin. Referring to the ventricular myocardial potential for the earliest activation mapping in PVCs resulted in a longer operation and fluoroscopy time. This suggests that the failure to map the FP in sinus rhythm cannot exclude PVCs originating from diseased fasicicles, and that the earliest FP should be sought during activation mapping.

### Case 2

**Fig. 3:**
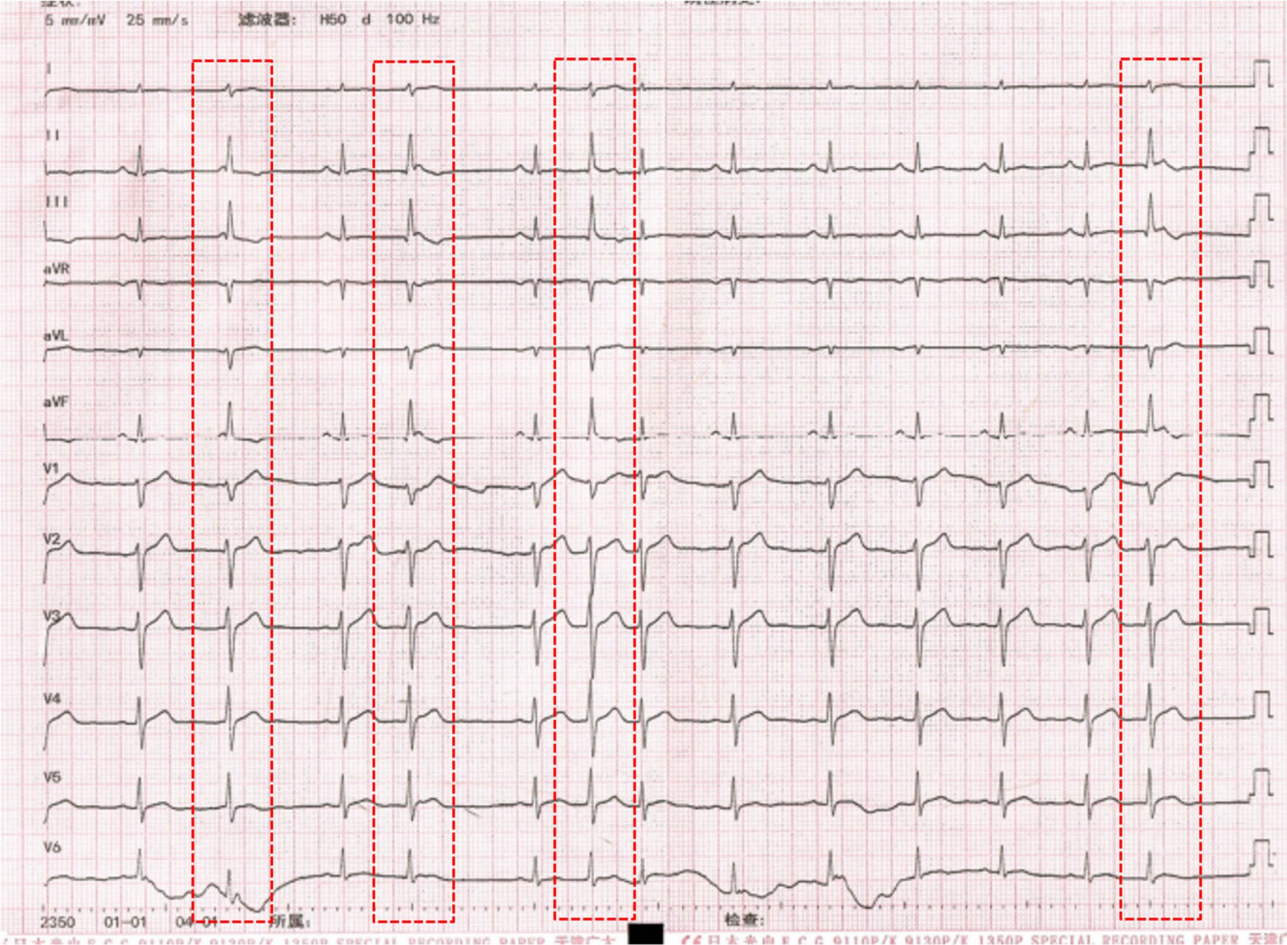
ECG shows a sinus rhythm with frequent PVCs. The red dashed box shows a PVC

**Fig. 4:**
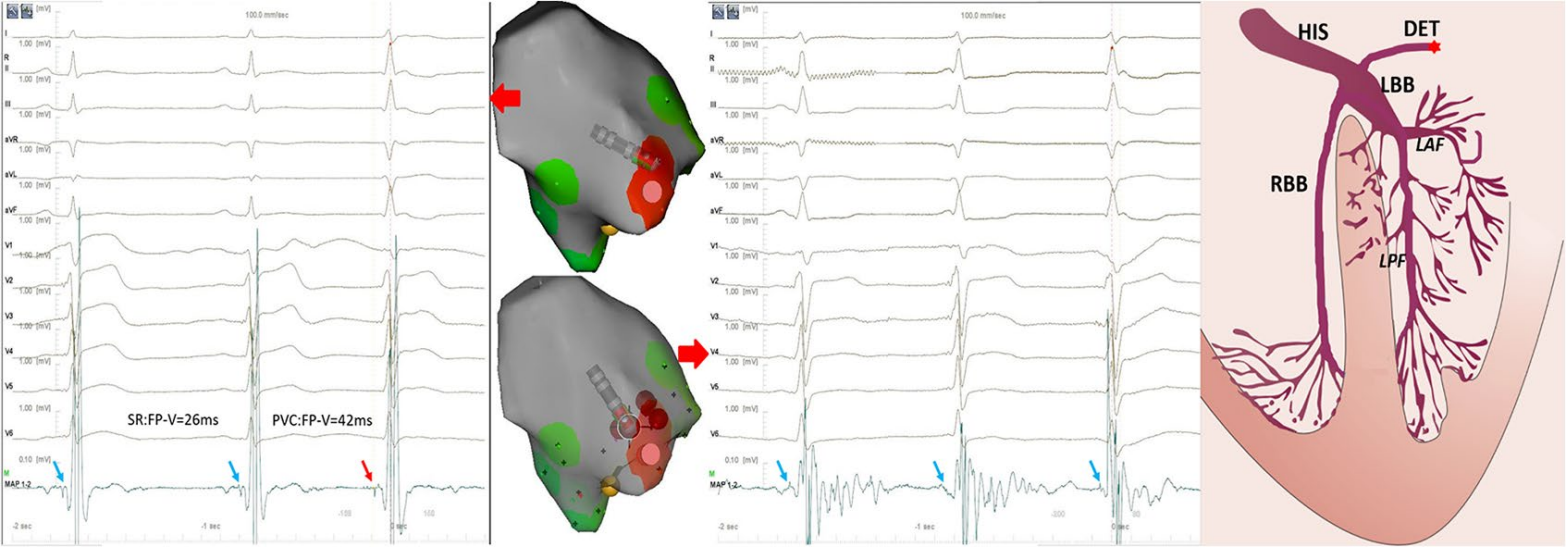
Electro-anatomical activation mapping of the PVCs and model diagrams of origin site of PVCs. Left panel: target electrogram shows FP (blue arrow) with FP-V interval of 26 ms in sinus rhythm and the earliest FP (red arrow) with FP-V interval of 42 ms in PVCs. Middle panel: RF energy application inducing Purkinje automaticity (blue arrow) with identical QRS morphology to the PVC. Right panel: model diagram of DET, the red star representing the origin location of PVCs

A 38-year-old man with prior medical history was referred for symptomatic frequent PVCs. The 24-h Holter ECG showed PVCs of 28,010 and the QRS duration of the PVCs was 97 ms. The baseline 12-lead ECG showed frequent PVCs with a normal axis and transition at V4. The QRS morphology of the PVCs was characterized by a deeper S wave in lead I, more apparent Q wave, higher R wave in inferior leads, and a lower S wave in lead V1 compared to sinus rhythm (Fig. 3). Based on the QRS wave pattern, it was presumed to have originated from the junction of the left bundle branch (LBB) with the left anterior fascicle (LAF). Activation mapping was performed in the aortic sinus of Valsalva during PVCs. At the right coronary cusp, a sharp presystolic low-amplitude and high-frequency potential that preceded the beginning of the V wave with isoelectric line between them was recorded at the earliest activation site. The pace-mapping failed to capture the potential and it preceded the QRS onset by 42 ms in PVCs and by 26 ms in sinus rhythm, where RF energy was delivered and induced Purkinje automaticity exhibiting a QRS morphology almost identical to that of the PVC. Subsequently, the PVCs were successfully eliminated (Fig. 4). The target site was at a distance of 14.2 mm from the His bundle recorded below the junction of the right coronary cusp and non-coronary cusp. Following completion of the lesion, over a waiting period of 30 min with isoproterenol infusion, no further PVCs were observed. The 24-h Holter monitoring revealed no PVCs after 3 months. De Vries L et al. reported that the DET was an anterior continuation of the specialized cardiac conduction system and was found at the base of the aortic sinuses, which is reminiscent of the continuation of the atrioventricular conduction ring that would have arrhythmogenic potential. The proximal end of the tract connects to the distal LBB or the proximal LAF, and the distal end is not electrically connected to the ventricular myocardium [5]. In this patient, the premature contraction activates the HPS and the ventricular myocardium by reverse conduction to the distal LBB or the proximal LAF, resulting in an FP-V interval in sinus rhythm at the earliest activation site shorter than that of PVCs. Therefore, the DET instead of the proximal LAF or distal LBB seemed to be the true origin of the PVCs in this case.

### Case 3

**Fig. 5:**
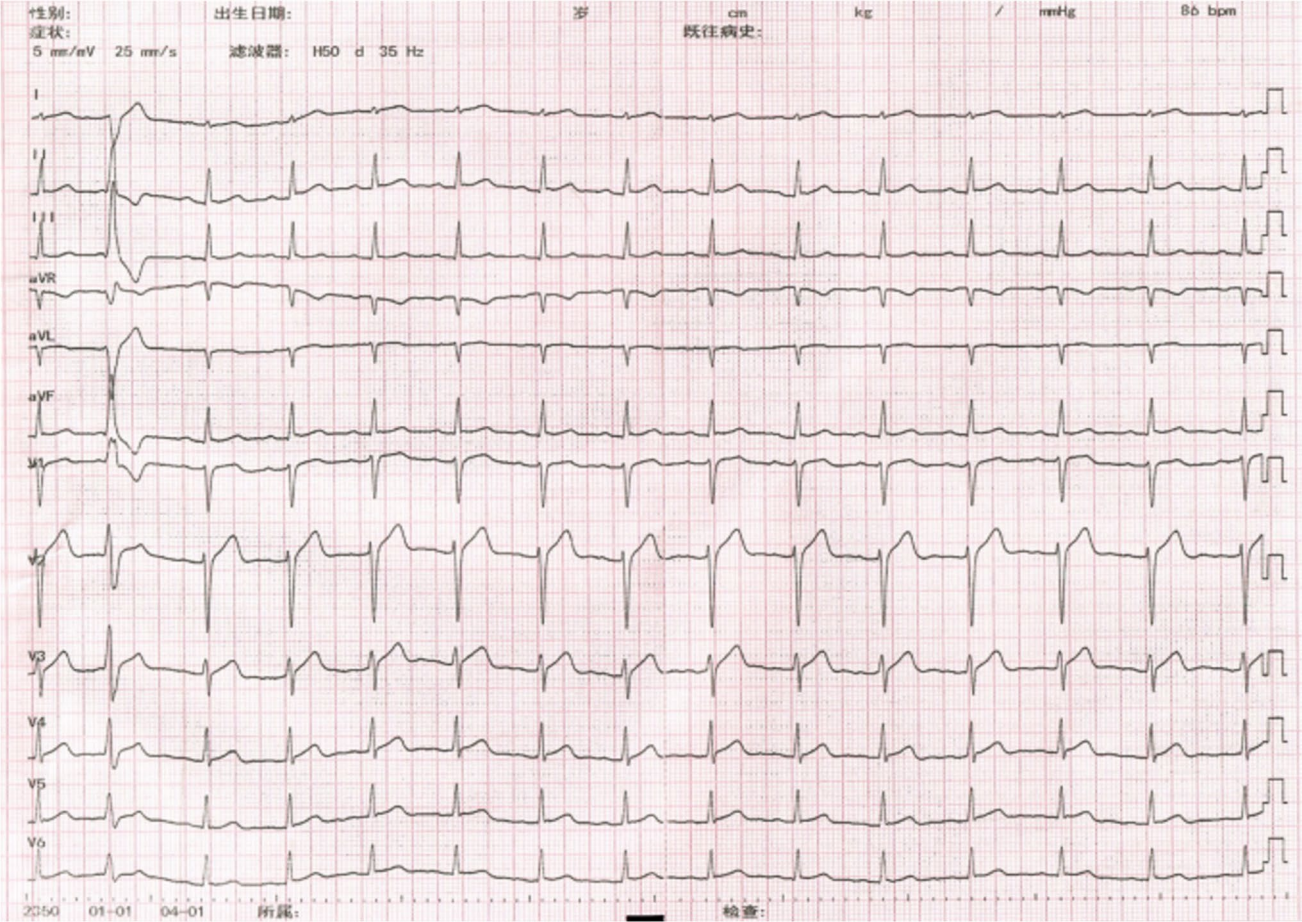
ECG indicates a sinus rhythm and PVCs

**Fig. 6:**
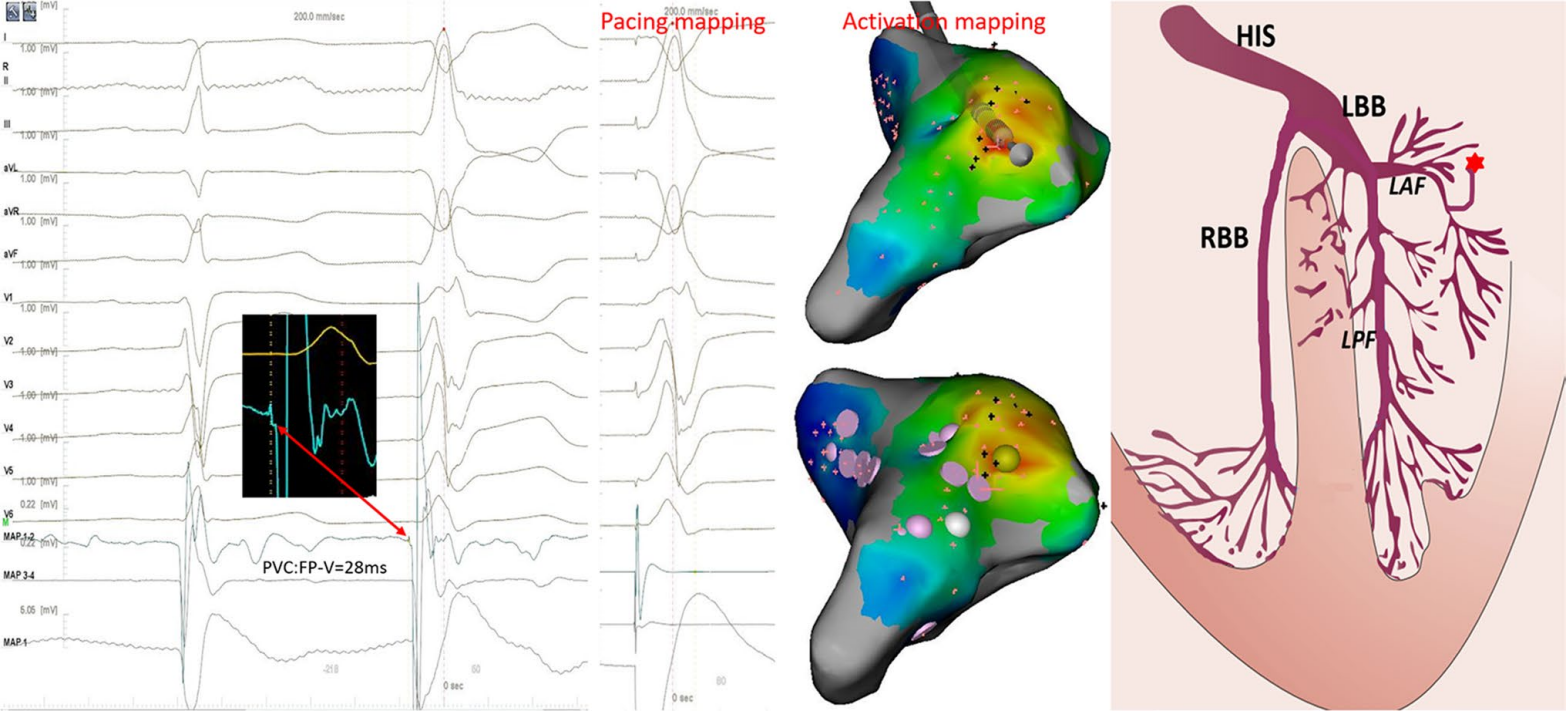
Electro-anatomical mapping of the PVCs and model diagrams of the origin site of PVCs. Left panel: activation mapping shows the earliest FP (red arrow) with FP-V interval of 28 ms, pace mapping shows the PASO score (PASO™, Biosense Webster) of 0.946. Right panel: model diagram of the mechanism without FP in sinus rhythm, the red star representing the origin location of PVCs

A 40-year-old woman was referred to our hospital and requested catheter ablation of symptomatic frequent PVCs after ineffective treatment with anti-arrhythmic medication and recurrent symptoms. The 24-h Holter ECG showed PVCs of 17,507 and the QRS duration of the PVCs was 128 ms. After admission, baseline 12-lead ECG showed a RBBB and inferior frontal plane QRS axis (Fig. 5). The LAF FP in sinus rhythm was mapped to construct a model of the anatomical distribution of the LAF. A very low-amplitude and high-frequency presystolic FP could be recorded in PVCs but not in sinus rhythm at the earliest activation site, which was very close to the distal LAF region during sinus rhythm. According to these findings, we determined that the PVC originated from the distal Purkinje system of the LAF, not from the myocardium. This presystolic FP preceded the earliest QRS onset by 28 ms. Pace mapping demonstrated an excellent match, with minor differences in inferior leads (Fig. 6). PVCs were successfully eliminated by RF energy delivery at this zone. Following completion of the lesion, no PVCs were observed with isoproterenol infusion and ECGs. Furthermore, the 24-h Holter monitoring after 3 months following the procedure revealed no PVCs. In this case, the electrograms of successful PVC ablation sites showed a presystolic low-amplitude and sharp potential, suggesting the activation of the end of the LAF due to the Purkinje fibers forming a complex and dense network covering the endocardium [6]. The earlier activation far-field potential of adjacent ventricular muscles may have preceded the local distal FP and resulted in failure of sinus rhythm mapping. The pace mapping at this location showed that the QRS morphology was very similar to that of the PVCs. It was useful at the distal HPS because it captured the FP and directly excited the ventricular myocardium.

## Discussion

The major methods for mapping PVCs include activation mapping and pace mapping. PVCs originate from the cardiac conductive system, which consists of bundles and fascicles, and the earliest presystolic Purkinje potential was a valid target for catheter ablation. The strategies of mapping PVCs originating from the HPS include the following: (1) mapping the earliest presystolic FP of PVCs, (2) predicting the FP-V interval at the target site. The earliest FP-V interval at the successful ablation site was similar to half of the additive value of the His-ventricular (HV) interval during SR and PVC [7]; (3) comparing the FP-V interval during SR and PVCs. The FP-V interval in sinus rhythm is equal to the FP-V interval of PVCs at the earliest FP activation. If the FP-V interval in sinus rhythm is greater than the FP-V interval in PVCs, this suggests that the location of origin of the PVCs is at the distal end of the mapping electrode [3].

In our cases, the presystolic FP was testified to be safe and effective for RFCA of PVCs originating from the HPS. In cases 2 and 3, the earliest activation of presystolic FP was recorded in PVCs and showed very tiny and sharp morphology features. The morphology of these low-amplitude and high-frequency potentials may be of importance in targeting the earliest activation sites of PVCs originating from the DET and the distal HPS. In addition, comparing predicted FP-V intervals with SR and PVC intervals is not of significant utility in our patients. Ventricular depolarization begins with action potentials that are propagated down the left and right bundle branches on either side of the ventricular septum. The left side of the septum is the first to depolarize owing to quicker conduction velocities of the left posterior branch, which results in the earliest onset of the QRS deflection [8]. As for PVCs originating from the normal proximal to middle left posterior fascicle, it is reasonable that the HV interval in sinus rhythm and HV interval in PVCs are used to infer the FP-V interval. Similarly, the FP-V value in sinus rhythm on the target electrogram being equal to the FP-V value in PVCs applies to these PVCs. During mapping, these methods are subject to errors for PVCs originating from diseased fascicles, DET, and distal fascicles.

Jinglin Z et al. reported that PVCs arose from the left fascicular system, and the earliest FP potential could be recorded in both sinus rhythm and PVC at the site of origin, which provides a definitive diagnosis of PVCs originating from the HPS [3]. In our study, FP could be recorded in PVC but not in sinus rhythm in cases 1 and 3. In case 1, the PVCs originated from the diseased fascicle, so it was impossible that the FP was recorded in sinus rhythm for the local ventricular activation preceding the diseased fascicle activation. In case 3, the PVCs originated from the distal fascicles, so it was sometimes difficult to use an ablation catheter to map these tiny potentials in some regions without FP distribution in sinus rhythm. According to these findings, it is not clear whether the PVCs originate from the fascicles, leading to misinterpretation of the PVCs as non-fascicle origin and activation mapping by the earliest ventricular activation. The simple adoption of mapping the earliest ventricular myocardial activation could lead to failure of catheter ablation or prolonged operation time.

The DET is part of the HPS and is a stump of the extended branch of the atrioventricular conduction loop, which links proximally to the distal LBB or the proximal LAF, with a structure that is insulated from the surrounding myocardium [9]. The PVC activation from here could only be conducted retrogradely to the insertion of the proximal segment of the conduction system [10]. In case 2, the morphological features of the PVCs are easily misinterpreted as having proximal LAF and distal LBB origin, and ablation at these regions may potentially increase the risk of atrioventricular block or left bundle branch block. The true origin for these so called “proximal LAF and proximal LBB” PVCs might be the DET. A precise grasp of the DET anatomy and electrophysiological characteristics and the detailed mapping of an earliest low-amplitude FP of the DET in the right coronary cusp or in the left ventricle beneath the valve can help prevent incorrect, high-risk ablation at the proximal LAF and distal LBB.

Some studies report pace mapping might be ineffective in identifying the target site of PVCs originating from bundles and fascicles. Since the pacing threshold of fascicles was usually higher than the ventricular myocardium, it is difficult to selectively capture local Purkinje fibers only, without activating the surrounding ventricular myocardium [3]. In case 3, the PVC origin was located at the distal fascicle, which connected electrically with the neighboring ventricular myocardium. The ectopic activation can not only directly excite the neighboring ventricular myocardium but also simultaneously conduct retrogradely to the proximal fascicle from where it activated the entire HPS. It is worth thinking that pacing will simultaneously activate local Purkinje fibers and the neighboring ventricular myocardium, resulting in complex pacing QRS morphology that might be totally similar to the clinical PVC itself. Pace mapping could be utilized to guide ablation of the idiopathic origin of PVCs from the terminal of the HPS.

In conclusion, our study analyzed the different intracardiac electrophysiological characteristics of PVCs originating from diseased fascicles, the distal His-Purkinje system, and the DET. Ablation of these PVCs can be safely and effectively performed by identifying the earliest FP.

## Data Availability

Data sharing is not applicable to this article as no new data were created or analyzed in this study.
